# The effect of ambient particle matters on hospital admissions for cardiac arrhythmia: a multi-city case-crossover study in China

**DOI:** 10.1186/s12940-018-0404-z

**Published:** 2018-07-16

**Authors:** Qiwen Zheng, Hui Liu, Jun Zhang, Dafang Chen

**Affiliations:** 1 Department of Epidemiology and Biostatistics, School of Public Health, Peking University, No.38 Xueyuan Road, Haidian District, Beijing, 100191 China; 2 Medical Informatics Center, Peking University, No.38 Xueyuan Road, Haidian District, Beijing, 100191 China; 3 Department of Neurology, Peking University People’s Hospital, No.11 South Xizhimen Street, Xicheng District, Beijing, 100044 China

**Keywords:** Air pollution, Particle matter, Cardiac arrhythmia, Hospitalization, Case-crossover

## Abstract

**Background:**

The relationship between particle matters (PMs) and cardiac arrhythmia has been investigated in numerous studies. However, evidence from developing countries is limited. The aim of this study was to evaluate the association between ambient PMs and hospital admissions for cardiac arrhythmia in China and to examine the potential effect modifiers.

**Methods:**

A time-stratified case-crossover analysis was conducted in 26 large Chinese cities. In total, we identified 175,265 hospital admissions for cardiac arrhythmia between January 2014 and December 2015 from electronic hospitalization summary reports. Conditional logistic regression was performed to estimate the percentage changes in cardiac arrhythmia admissions in relation to interquartile range increases in air pollutants. Age, gender and prespecified comorbid health conditions including hypertension, diabetes, congestive heart failure and hyperlipidemia were stratified to evaluate susceptibility factors.

**Results:**

PMs levels were positively associated with the number of hospital admissions for cardiac arrhythmia. Both PM_2.5_ and PM_10_ had the strongest impact on lag 2 days. An interquartile range increase in PM_2.5_ (47.5 μg/m^3^) and PM_10_ (76.9 μg/m^3^) concentrations on lag 2 days was associated with increments of 2.09% (95%CI, 1.58–2.60%) and 2.33% (95%CI, 1.68–2.97%) in hospital admission for cardiac arrhythmia, respectively. Evidence of effect modification by age and comorbid diabetes was observed. The elderly (> 65 years) and patients with comorbid diabetes were more likely to be hospitalized for cardiac arrhythmia following exposure to high levels of PMs.

**Conclusions:**

This study found an increased risk of arrhythmia admissions associated with PM_2.5_ and PM_10_ levels among 26 Chinese cities. The associations of PMs with arrhythmia admissions were stronger in aged population and people with diabetes.

**Electronic supplementary material:**

The online version of this article (10.1186/s12940-018-0404-z) contains supplementary material, which is available to authorized users.

## Background

Over the past decades, with the development of industry and economy, air pollution contributes to a considerable public concern worldwide, especially in developing countries, in which ambient particle matters (PMs) were considered as the predominant pollutant. Ambient PMs was the fifth-ranking mortality risk factor in 2015, accounting for 7.6% global deaths and 4.2% DALYs [1]. In China, it was ranked the forth risk factor for disease burden, leading to more than 0.9 million premature deaths annually [2].

Cardiac arrhythmia consists of a group of complex conditions which is related to the risks of cardiovascular complications and sudden deaths [3]. Increasing evidence revealed the facts that air pollution is associated with the risk of hospital admission, emergency room visits and mortality from cardiovascular causes [4–7]. Deeply knowing the association between PMs and specific cardiovascular endpoint is proving of great importance to better understand the physio pathological mechanisms, aiding the development of personalized interventions and the making of healthcare policies.

Several epidemiological studies have suggested that exposure to ambient particle matters might be responsible for cardiac arrhythmia [8–11]. Recently, a meta-analysis also demonstrated the temporal association between PM_2.5_, PM_10_ and arrhythmia hospitalization or mortality with the risk ratio 1.015 per 10 μg/m^3^ and 1.009 per 10 μg/m^3^, respectively [12]. However, most of the previous studies which assessed the association between particle matters and cardiac arrhythmias were carried out in North America, Western Europe and East Asia. These findings may not be applicable to Mainland of China, because of the different ambient air pollutant mixtures, weather patterns, health status and population susceptibility. Furthermore, although one study has recently been conducted in China to explore the relationship between air pollution and cardiac arrhythmia [13], the evidence was still limited in China as data were often restrained from individual cities, introducing uncertainties to results in external validation. In addition, evidence from previous studies has shown that persons with comorbidities may be at increased risk of cardiovascular morbidity and mortality [14, 15], but little is known about the potentially sensitive groups in Chinese populations.

Therefore, the primary objective of this study was to explore the association between ambient particle matters and hospital admissions for cardiac arrhythmia in 26 large Chinese cities during 2014–2015, by using a time-stratified case-crossover design. The secondary objective was to examine whether subjects with specific comorbid health conditions were more susceptible to particle matters.

## Methods

### Study design

A time-stratified case–crossover study were performed to investigate the association between PMs and daily cardiac arrhythmia hospital admissions [16]. In this approach, the cases played a role as their own controls, where the exposure experience at different time periods (before or after the case period) were compared. For each case of cardiac arrhythmia, the control days were chosen on a fixed time stratum, typically the days falling in the same city on the same day of week and within the same calendar month as the index day (the day of hospitalization for cardiac arrhythmia). By using this study design, precise control for long-term trend, day of week and some other slowly changing individual-level factors such as age, gender, race, smoking status, educational background and lifestyles were achieved.

### Hospital admissions data

The daily hospital admissions for cardiac arrhythmia in 26 cities during the study period were obtained from electronic hospitalization summary reports (HSRs) of the top-ranked hospitals (Grade 3A) in care, safety and quality as evaluated by the National Hospital Performance Project of the National Healthcare Data Center of China. More than 15 million HSRs were collected for this project during 2014 to 2015. The detailed description of this project has been published previously [17]. The standard HSRs includes information on basic demographics, dates of admission and discharge, hospitalization diagnoses (one principle diagnosis and ten comorbidities) and their corresponding International Classification of Diseases, 10th Revision (ICD-10) codes, treatments, outcomes of hospitalization and financial costs.

The primary outcome of our study is cardiac arrhythmia daily admission counts. We used ICD-10 codes to identify arrhythmia-related admissions between January 1, 2014 and December 31, 2015 in each study city, and then transformed them into daily counts for further modeling analysis. These codes included I44 (atrioventricular and left bundle-branch block), I45 (other conduction disorders), I46 (cardiac arrest), I47 (paroxysmal tachycardia), I48 (atrial fibrillation and flutter), and I49 (other cardiac arrhythmia). Patients aged less than 18 years old were excluded from the present study. In total, 175,265 hospitalization admissions were identified from 26 large cities (shown in Additional file 1: Figure S1) in China during the study period.

Age, gender and prespecified selected comorbid health conditions were the effect modifiers of interest. ICD-10 codes were used to identify the comorbidities in addition to a primary diagnosis of cardiac arrhythmia for each subject. The comorbidities examined in this study were defined as follows: diabetes mellitus (E10-E14), hypertensive diseases (I10-I13, I15), heart failure (E50) and hyperlipidemia (E78).

### Environmental data

Daily average concentrations of air pollutants in 26 Chinese cities were obtained from the National Urban Air Quality Real-time Publishing Platform (http://106.37.208.233:20035/, with detailed description in Additional file 1). In brief, the system fulfills the quality assurance and quality control mandates of the Chinese government through its ambient air-monitoring stations. These stations provide hourly air pollution data to the system. The air pollutants in this study included particulate matter with an aerodynamic diameter less than 2.5 μm (PM_2.5_), particulate matter with an aerodynamic diameter less than 10 μm (PM_10_), carbon monoxide (CO), nitrogen dioxide (NO_2_) and sulfur dioxide (SO_2_). The daily (24-h) mean concentrations of pollutants averaged across all the stations in a given city were used as the reading for that city on that day. In addition, meteorological data including 24-h average temperature (°C) and relative humidity (%) for each city in the study were obtained from Chinese Meteorological Bureau.

### Statistical analysis

All statistical analyses were conducted using R (V.3.3.3, R Development Core Team). Data are presented as mean ± SD, median (interquartile range), or absolute number and percentages, as appropriate. Spearman’s correlation analyses were performed to examine the relationship between air pollutant variables. Conditional logistic regression was used to explore the associations between PM and cardiac arrhythmia. The distributed lag non-linear models with three degrees of freedom in the natural cubic splines and a maximum lag of 3 days were applied to adjust the delayed and non-linear effects of temperature and humidity [18]. Interactions between meteorology and cities were included in the model to control possible confounding factors, including baseline prevalence of cardiac arrhythmia and weather conditions in each city. Public holidays were also incorporated. Both single-day lag structure (Lag0 to Lag5) and multiple-day lag structure (Lag0–2, Lag0–3, Lag0–4 and Lag0–5) were employed in this analysis to examine the temporal association between PMs and arrhythmia. In addition, a smoothing function was applied to graphically analyze the exposure–response association between PMs concentration and cardiac arrhythmia hospitalizations.

In order to examine potential confounding factors by other air pollutants, two-pollutant models adjusted for CO, NO and SO_2_ were applied. Stratification analyses by age, gender and prespecified comorbidities to explore the potential individual level effect modifiers of particle matters were also conducted using the above models. Subgroup analyses were compared using a Z-test [19]. The results are expressed as the percentage change and 95% confidence interval (CI) in the daily cardiac arrhythmia hospital admissions per interquartile range (IQR) increase in PMs concentration. The reported significance levels were all two-sided, with statistical significance set at 0.05.

## Results

A total of 175,265 patients whose principle diagnosis was cardiac arrhythmia during their hospitalization were included in this study. The basic characteristics of the study population were listed in Table 1. The median age was 59.0 (IQR: 48.0–70.0) years old with 50.6% male patients. Among all the prespecified comorbid health conditions, hypertension was the largest, with 37% of the admissions for cardiac arrhythmia having a comorbidity of hypertension. Other comorbid health conditions included were diabetes mellitus, hyperlipidemia and congestive heart failure, with 11.4, 13.7 and 10.6% of total hospital cardiac arrhythmia admission, respectively. The descriptive statistics for the cardiac arrhythmia hospital admissions, air pollutants and meteorological variables were listed in Table 2. There was an average of 240 daily hospital admissions for cardiac arrhythmia in the 26 Chinese cities across the study period. The mean particle matters level during the study period was 106.8 μg/m^3^ for PM_10_ and 63.5 μg/m^3^ for PM_2.5_. Mean daily concentrations of SO_2_, NO_2_ and CO were: 29.6 μg/m^3^, 44.1 μg/m^3^ and 1.15 mg/m^3^, respectively. Spearman’s correlation analyses showed that the concentrations of PM_2.5_ and PM_10_ were highly correlated (Table 3, *r* = 0.87). Meanwhile, SO_2_, NO_2_ and CO were moderately correlated with PM_2.5_ and PM_10_ (*r* = 0.61–0.68).

**Table 1:** Characteristics of the study population (*n* = 175,265)

	No. (%)
Demographic	
Gender	
Male	88,760 (50.6%)
Female	86,505 (49.4%)
Age	
< 65 years old	112,403 (64.1%)
≥ 65 years old	62,862 (35.9%)
Comorbid health condition	
Hypertension	
With	64,786 (37.0%)
Without	110,479 (63.0%)
Diabetes	
With	19,947 (11.4%)
Without	155,318 (88.6%)
Hyperlipidemia	
With	23,957 (13.7%)
Without	151,308 (86.3%)
Congestive heart failure	
With	18,529 (10.6%)
Without	156,736 (89.4%)

**Table 2:** Distribution of daily cardiac arrhythmia hospital admissions, meteorological data and air pollutants in 26 Chinese cities, 2014–2015

Variable	Mean ± SD	Minimum	Percentile			Maximum	IQR
			25th	50th	75th		
PM_2.5_ (μg/m^3^)	63.5 ± 50.6	5.1	31.5	49.4	79.0	897.5	47.5
PM_10_ (μg/m^3^)	106.8 ± 71.9	7.4	58.3	89.4	135.2	977.3	76.9
SO_2_ (μg/m^3^)	29.6 ± 32.6	1.9	11.4	18.8	33.6	316.9	22.2
NO_2_ (μg/m^3^)	44.1 ± 19.4	4.5	30.0	40.2	54.1	175.8	24.1
CO (mg/m^3^)	1.15 ± 0.63	0.14	0.76	0.99	1.32	8.41	0.56
Temperature(°C)	14.5 ± 10.9	−25.7	7.0	16.4	23.3	35.5	16.3
Relative humidity (%)	69.2 ± 33.2	8	53	69	80	97	27
Daily arrhythmia admissions	240.1 ± 129.8	2	93	270	320	555	227

*SD* standard deviation, *IQR* interquartile range

**Table 3:** Correlation coefficients among air pollutants

Variables	PM_2.5_	PM_10_	NO_2_	SO_2_	CO
PM_2.5_	1.00	0.87*	0.67*	0.61*	0.68*
PM_10_	―	1.00*	0.64*	0.63*	0.60*
NO_2_	―	―	1.00	0.54*	0.59*
SO_2_	―	―	―	1.00	0.55*
CO	―	―	―	―	1.00

*P < 0.001

**Fig. 1:**
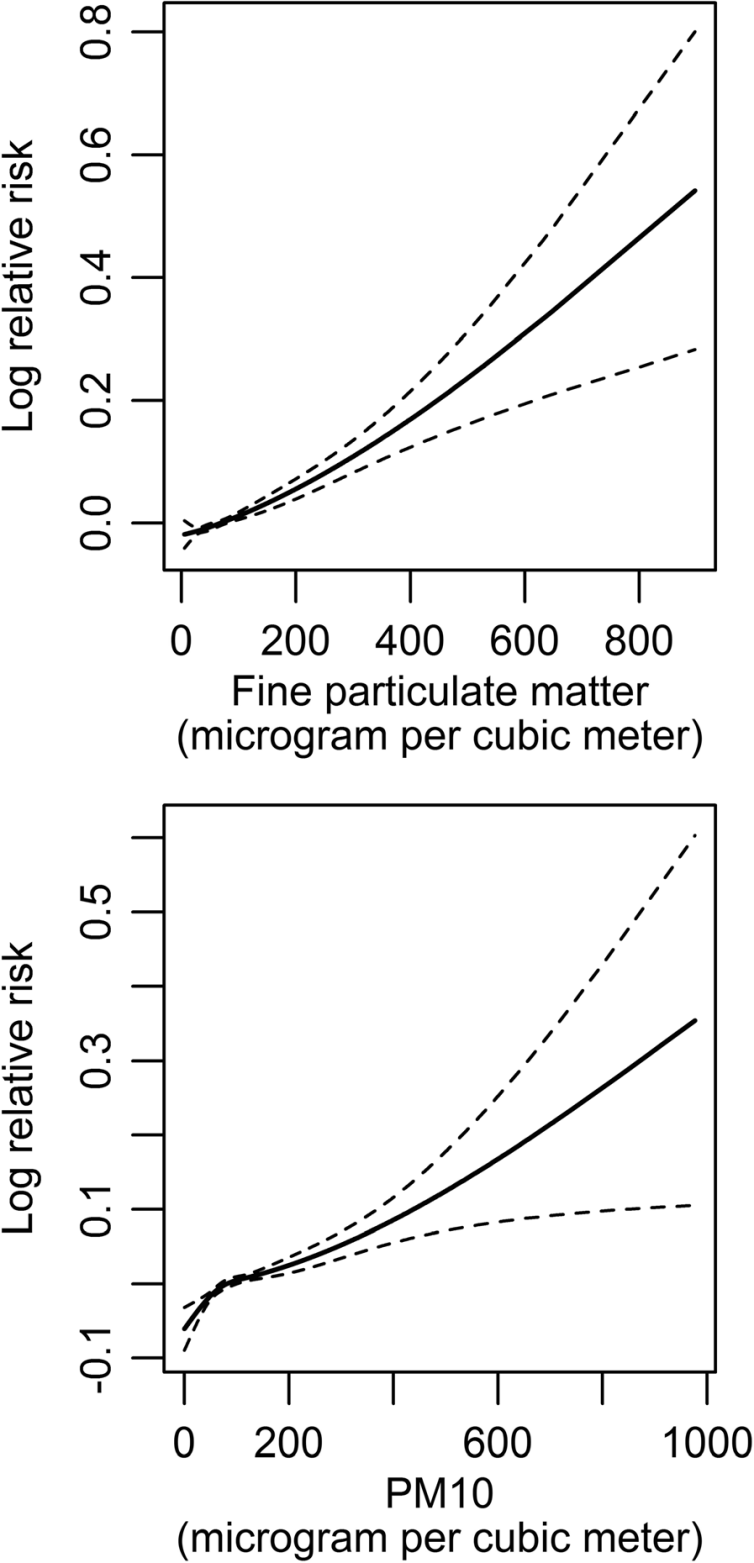
Dose-response relationship between cardiac arrhythmia hospital admissions in 26 Chinese cities and particle matters concentration (degree of freedom = 3). The x-axis of the upper part is the 4-d (lag0–3) moving average concentration of fine particulate matter (μg/m^3^); the x-axis of the lower part is the 5-d (lag0–4) moving average concentration of PM_10_ (μg/m^3^). The y-axis is the predicted log (relative risk) after adjusting for the temperature, relative humidity, public holidays and city-level confounders. The solid lines represent the effect estimates and the dashed lines represent 95% confidence intervals

Figure 1 depicted the exposure-response curves for daily concentration of PM_2.5_ and PM_10_ with hospitalization for cardiac arrhythmia. The concentration-response relationship appeared to be linear and positive without any thresholds. Table 4 presented the percentage change in daily admission for cardiac arrhythmia associated with an IQR increase in particle matters concentrations for different lag structures. For PM_2.5_, the single-lag model estimated positive and significant associations on lag 0, 1, 2 and 3 days. Consistent results were observed between PM_10_ and cardiac arrhythmia. Both PM_2.5_ and PM_10_ had the strongest impact on lag 2 days. An IQR increment in PM_2.5_ and PM_10_ concentrations on lag 2 days was associated with increments of 2.09% (95%CI, 1.58–2.60%) and 2.33% (95%CI, 1.68–2.97%) in hospital admission for cardiac arrhythmia, respectively. The cumulative increase for arrhythmia admission due to PM_2.5_ was 2.93% across three days, and the effect due to PM_10_ was 3.58% across four days. An IQR increase in NO_2_ and CO concentrations on lag2 day corresponded with a 2.45% (95%CI, 1.73–3.17%) and 2.79% (95%CI, 2.20–3.39%) increases in cardiac arrhythmia admissions, respectively. However, the association between SO_2_ and cardiac arrhythmia admissions is insignificant (− 0.80%, 95CI, − 1.42 - 0.18%, *p* = 0.066).

**Table 4:** Percentage change with 95% CI in cardiac arrhythmia admissions associated with an IQR increases in PM_2.5_ (47.5 μg/m^3^) and PM_10_ (76.9 μg/m^3^) concentration for different lag structure

Lag Days	PM_2.5_			PM_10_		
	Percentage Change	95% CI	*p* value	Percentage Change	95% CI	*p* value
Lag 0 days	1.50	0.98–2.02	< 0.001	1.56	0.92–2.19	< 0.001
Lag 1 days	1.89	1.37–2.40	< 0.001	1.91	1.27–2.55	< 0.001
Lag 2 days	2.09	1.58–2.60	< 0.001	2.33	1.68–2.97	< 0.001
Lag 3 days	1.48	0.97–2.00	< 0.001	2.15	1.52–2.79	< 0.001
Lag 4 days	0.00	−0.52 - 0.52	0.997	0.65	0.02–1.28	0.043
Lag 5 days	−0.36	−0.88 - 0.16	0.177	−0.10	−0.73 - 0.54	0.767
Lag 0–2 days	2.67	2.03–3.31	< 0.001	2.92	2.11–3.74	< 0.001
Lag 0–3 days	2.93	2.24–3.63	< 0.001	3.56	2.67–4.46	< 0.001
Lag 0–4 days	2.67	1.91–3.43	< 0.001	3.58	2.61–4.56	< 0.001
Lag 0–5 days	2.28	1.47–3.09	< 0.001	3.22	2.19–4.26	< 0.001

*CI* confidence interval, *IQR* interquartile range. The association was adjusted for temperature, relative humidity, public holidays and city-level confounders

In the two-pollutant models (Table 5), the effect of PM_2.5_ and PM_10_ on cardiac arrhythmia admissions was slightly stronger when controlling for SO_2_. PM_2.5_ showed consistent and significant associations with arrhythmia when adjusted for CO and NO_2_. Nevertheless, the association of arrhythmia admissions with PM_10_ was insignificant and even close to null after controlling for NO_2_ and CO.

**Table 5:** Estimated odds ratio and 95% confidence intervals in cardiac arrhythmia admissions associated with air pollutants concentrations on lag 2 day in two-pollutant models

Variable	PM_2.5_	PM_10_
Adjusted for NO_2_	0.80(0.14–1.47)*	− 0.08(− 0.91–0.76)
Adjusted for SO_2_	3.29(2.71–3.87)***	3.35(2.62–4.09) ***
Adjusted for CO	0.95(0.24–1.66)**	0.27(−0.57–1.11)

*P < 0.05 ** *P* < 0.01 *** *P* < 0.001

**Fig. 2:**
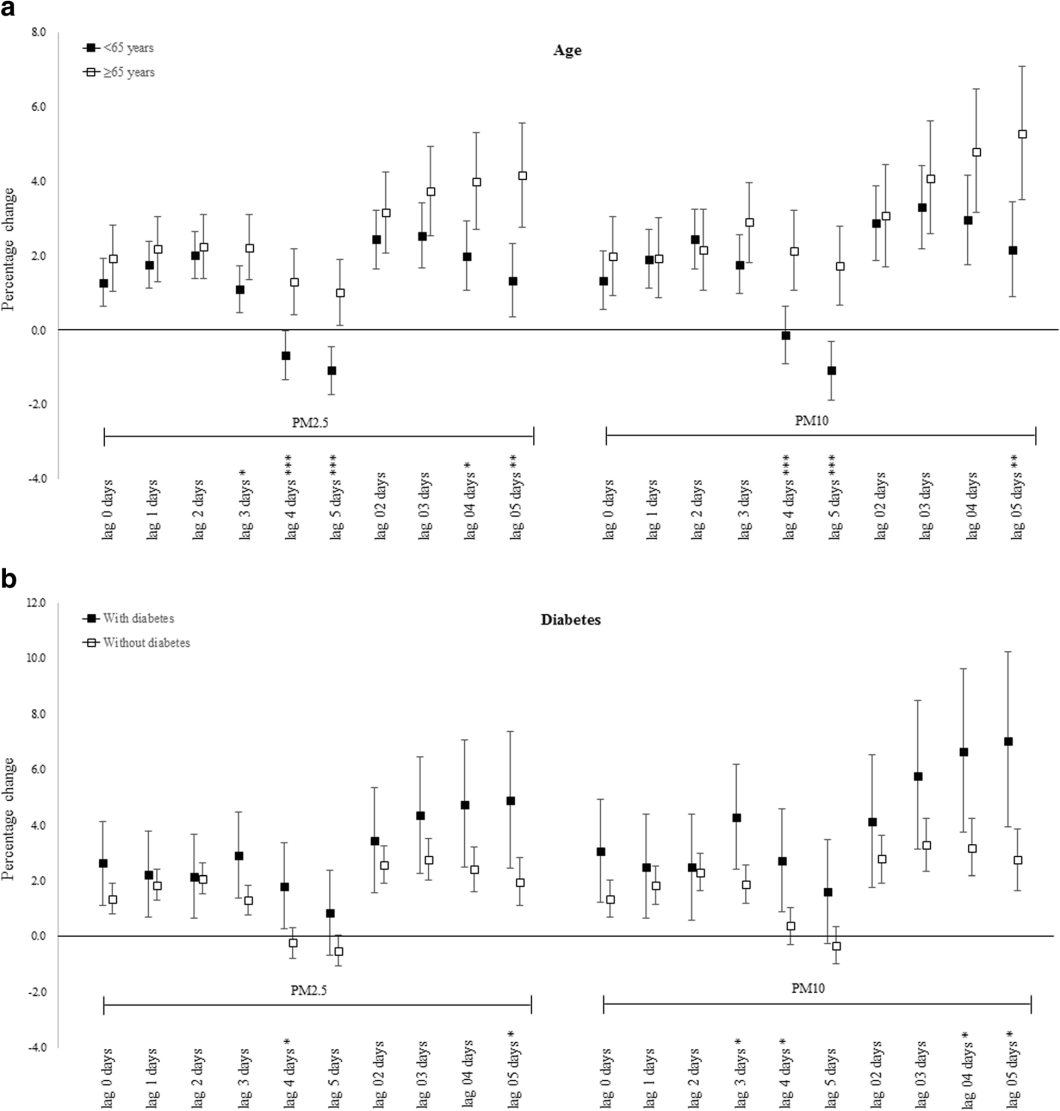
Percentage change with 95% confidence interval in cardiac arrhythmia admissions associated with an interquartile range increase in PM_2.5_ (47.5 μg/m^3^) and PM_10_ (76.9 μg/m^3^) concentrations stratified by age (**a**) and diabetes (**b**). Note: *P < 0.05 ** *P* < 0.01 *** *P* < 0.001

In the subgroup analysis, we examined the age, gender and concurrent comorbid health conditions including hypertension, diabetes, hyperlipidemia and congestive heart failure as potential effect modifiers. Figure 2a shows the associations between PM concentrations and hospitalizations for arrhythmia, stratified by age. The association for both PM_2.5_ and PM_10_ were stronger and lasted longer in the elderly. An IQR increase in PM_2.5_ and PM_10_ concentrations on lag5 day resulted in 1.01% (95%CI, 0.13–1.89%) and 1.72% (95%CI, 0.65–2.79%) increases, respectively, in admissions among the elderly, compared with the null or even negative association in adults aged 18–65. The differences between the two groups were statistically significant (PM_2.5_: *p* < 0.001; PM_10_: *p* < 0.001). Analyses revealed a significant association between exposure to air pollutants and hospitalizations for arrhythmia in individuals with diabetes. An IQR increase in PM_2.5_ and PM_10_ concentrations on lag4 day resulted in 1.80% (95%CI, 0.28–3.35%) and 2.73% (95%CI, 0.89–4.59%) increases, respectively, in hospitalization for arrhythmia in patients with diabetes, but only a − 0.24% (95%CI, − 0.79 - 0.32%) and 0.37% (95%CI, − 0.29 - 1.05%) increases in patients without diabetes (Fig. 2b). However, gender and all comorbidities other than diabetes failed to show a noticeable effect modification between the concentration of particle matters and arrhythmia admissions in any lag structures (all *p* > 0.05; shown in Additional file 1: Figure S2).

## Discussion

In our study, we took the advantage of a large database of hospital visits collected over 26 large Chinese cities during a two-year time period to examine the association between airborne particle matters and cardiac arrhythmia hospitalization. To the best of our knowledge, this is the first multisite study in China, or even in the Asia, to examine the effects of PMs pollution on arrhythmia. We observed that short-term elevated concentration of PM_2.5_ and PM_10_ were significantly associated with increased risk of cardiac arrhythmia hospital admissions. An IQR increase in PM_2.5_ and PM_10_ on lag 2 day associated with a 2.09% (0.44% for 10 μg/m^3^ increase in PM_2.5_) and 2.33% (0.30% for 10 μg/m^3^ increase in PM_10_) increase in admissions for cardiac arrhythmia, respectively. We did not find evidence of effect modification by gender, but we found a stronger and longer lasting effect among aged population and people with diabetes. Other prespecified comorbid health conditions including hypertension, congestive heart failure and hyperlipidemia did not modify the risk.

Panel studies [20, 21], which required participants to wear a Holter monitor or implantable cardioverter defibrillators (ICDs), have confirmed the association between air pollution episodes and cardiac arrhythmia. However, the results from studies exploring the relationship between air pollution and the risk of having arrhythmia-related hospitalization or ER visits among larger and more general populations remain controversial. In Canada [22], a time-series analysis conducted among nearly 400,000 ER visits in seven cities failed to demonstrate the association between arrhythmia and daily PMs concentrations. Another case-crossover study in the US [23] showed atrial fibrillation hospital admission is not increased with short-term elevation in exposure to PM_2.5_ either. On the contrary, there is a growing body of literature linking cardiac arrhythmia with exposure to airborne PMs and give positive conclusion. Talbott [24] investigated over 7,500,000 hospital admissions for circulatory diseases across seven states in the United States during 2001 to 2008, and demonstrated significant associations between ambient PM_2.5_ levels, after controlling for temperature and ozone, and arrhythmia hospitalizations, especially in the cooler months. In the UK [10], a large case-crossover study linking daily mean concentrations of air pollutants with three databases, the Myocardial Ischemia National Audit Project (MINAP), Hospital Episode Statistics (HES) and Mortality (Office for National Statistics), involving over 2 million CVD emergency hospital admissions and over 600,000 CVD deaths, demonstrated that PM_2.5_ is associated with a raised risk of arrhythmia hospitalization and mortality. In East Asia, Ueda [25] conducted a multicity study in Japan and found a significantly positive association between arrhythmia mortality and PM_2.5_. Agreeing with our finding, two recent studies performed in Taipei [26, 27] demonstrated the significant positive association between PM concentration and arrhythmia ER visits or hospital admissions. Recently, a time-series analysis study [13] including 56,940 outpatient visits for cardiac arrhythmia conducted in Shanghai, China, found that a 10 μg/m^3^ increase in the present-day concentration of PM_10_ corresponded to increase of 0.56% (95% CI, 0.42 to 0.70%) in outpatient arrhythmia visits. The inconsistency in results reported around the world may stem from the different concentration level of ambient particle matters, complex component of the pollutants, population susceptibility and so forth.

We have found that age modified the risk for arrhythmia hospitalization in relation to PM_2.5_ and PM_10_ concentration levels. This conclusion was generally consistent with most previous reports. Zhao [13] found that the associations between PM_10_ and outpatient arrhythmia visits were stronger in elderly participants. In MINAP study [10], the effect of PM_2.5_ on mortality was higher in those over the age of 70. The frailty of the elderly is the most likely reason why they show a strong reaction on a high level of PMs concentration. It is plausible that most elderly have higher prevalence of chronic cardiopulmonary diseases, leading to poor health conditions. Therefore, they are more susceptible to the adverse effect of particle matters. However, results concluded from other studies [8, 23] showed weak evidence for effect modification by age groups. Different data source, outcome definition and study design may interpret part of the reason and further studies are needed to clarify this inconsistency.

Evidence from epidemiological studies [28–30] reported that persons with chronic comorbid health conditions may be at increased risk of cardiovascular morbidity and mortality associated with air pollutants levels. Existing diabetes modified the association of particle matters and arrhythmia admissions have been demonstrated in some previous studies. For example, in Park’s study [31], persons with ischemic heart disease, hypertension or diabetes appear to be more susceptible to autonomic dysfunction related to PM_2.5_ exposure. Peel [14], by evaluating over 4 million emergency department visits from 31 hospitals in America, also found the estimated association of arrhythmia in relation to PM_10_ was substantially higher among patients with hypertension or diabetes than for patients without such comorbid conditions, whereas COPD and congestive heart failure provided little evidence of effect modification. Possible explanation is that exposure to particle matters is associated with reduced heart rate variability, increased C-reactive protein levels and elevated inflammatory markers, which shared the same pathway with diabetes. In contrast, Colais [15] identified a series of chronic conditions and failed to find a clear marker of susceptibility for arrhythmia on the effect of PM_10_. Bunch [23] observed no additive risk between PM_2.5_ and atrial fibrillation hospitalization in those with respiratory disease either. Inconsistency of results may derive from factors related to the disease itself. Patients with cardiovascular disease are more likely to reduce exposure to the air pollution and taking antihypertensive or heart rhythm control medications, thus reducing their occurrence of arrhythmia symptom.

Recent studies [32, 33] have provided a limited but growing understanding of possible biological mechanisms. One major hypothesis is the autonomic nerve system dysfunction, which results in increased heart rate and impaired heart rate variability. Such autonomic modulation triggers the onset of premature ventricular contractions and premature atrial contractions, and the lag structures found in most studies indicate a rapid pathway. In patient-based study, Rich [34–36] pointed out the evidence of acute effects (1 h) of elevated PMs on severe arrhythmias patients wearing ICDs. In large population-based study, Santos [37] examined 3251 arrhythmia ER visits and drew the conclusion that the association between arrhythmia and daily PM_10_ was acute and limited to the same day of exposure. Zhao [13] also reported the effect of PM_10_ concentration was limited within the concurrent day by analyzing 56,940 outpatient visits for arrhythmia in Shanghai. In our study, we found the results that both PM_2.5_ and PM_10_ had the strongest impact at lag 2 days, which is in line with other previously published studies [10, 26]. One potential explanation for this discrepancy in the different lag structure is that the use of hospital admissions rather than outpatient visits or emergency room visits. Delay in time is existed between symptom onset and hospital admission for further treatment. In consequence, the lag effects of particle matters on cardiac arrhythmia hospital admissions should be interpreted with caution.

The strengths of our investigation include the national coverage of a wide range of hospital admissions among 26 Chinese cities. Multicity studies have the advantage of generating more reliable results and tend to be less vulnerable to bias compared with small studies conducted at single site. To our knowledge, this is the first multicity study in China, or even in the Asia, to examine the short-term effects of air pollution on arrhythmia. An additional strength is the ability to detect comorbid health status. By using large amounts of clinical information, potential effect modifiers could be identified.

Some limitations need to be addressed. Firstly, as in most previous epidemiological studies, we have used the averaged monitoring results across various stations as the proxy for the population exposure level. This might bring about exposure measurement errors because personal exposure depends on a number of issues, such as daily outdoor activities, the use of air cleaner, location of residence and so forth. Another limitation of our study is the use of data from hospital admissions instead of outpatient visits. Some kinds of arrhythmia often show no symptoms. Mild cases who suffered palpitations or feeling a pause between heartbeats may choose to take medication at home or to visit their family doctors. Besides, due to the inclusion of top-ranked hospitals only, the characteristics of severity of illness and socio-demographic conditions of the cases included in our analysis might be different from those patients admitted into lower ranked hospitals. Therefore, the generalizability of our results should be interpreted with caution. Thirdly, comorbid health conditions were evaluated merely based on concurrent hospital discharge records rather than including diagnoses from previous visits, potentially reducing the sensitivity of comorbid conditions assessment and leading to misclassify of the subgroup. For this reason, the effect difference of airborne particle matters between groups with and without specific secondary conditions may tend to null. However, some studies [30] showed little or no difference in using concurrent or previous records when evaluating comorbidity. Moreover, two-pollutant models were applied to examine the robustness of the associations between PMs and cardiac arrhythmia. However, the collinearity among the pollutants added uncertainty to the interpretation of the results and limited our ability to isolate the independent effects of PMs on cardiac arrhythmia. Therefore, the results of two-pollutant models should be interpreted with caution. Finally, due to the limit information, the inability to differentiate all subtypes of cardiac arrhythmia is another limitation in our study. Nevertheless, both ventricular and supraventricular kinds of arrhythmia have been demonstrated [37] its susceptibility to the effect of air pollutants. Further studies are needed to confirm whether there is modification effect across different subtype of cardiac arrhythmia or not.

## Conclusion

In summary, by using a considerably larger sample of patients than previous studies in developing world, our study provided robust evidence that an increased risk of arrhythmia admissions was associated with the level of PM_2.5_ and PM_10_ among 26 large cities in China. Evidence of effect modification by age and comorbid diabetes was observed in response to PM levels. Future studies with tightly controlled exposure level, in addition to long-term observational studies are needed to help elucidate the effect in the long run.

## Additional file

Additional file 1: **Figure S1.** Locations of 26 Chinese cities and their average daily PM2.5 concentration during the study period. 26 Chinese cities included Harbin (71 μg/m3), Changchun (65 μg/m3), Shenyang (72 μg/m3), Dalian(50 μg/m3), Beijing (82 μg/m3), Tianjin (78 μg/m3), Shijiazhuang (106 μg/m3), Jinan (90 μg/m3), Zhengzhou (91 μg/m3), Xi’an (67 μg/m3), Lanzhou (54 μg/m3), Yinchuan (48 μg/m3), Xining (55 μg/m3), Urumchi (64 μg/m3), Chengdu (67 μg/m3), Chongqing (59 μg/m3), Wuhan (75 μg/m3), Changsha (67 μg/m3), Nanchang (46 μg/m3), Nanjing (65 μg/m3), Shanghai (53 μg/m3), Hangzhou (58 μg/m3), Kunming (31 μg/m3), Nanning (45 μg/m3), Guangzhou (43 μg/m3) and Fuzhou (30 μg/m3). **Figure S2.** Percentage change with 95% confidence interval in arrhythmia admissions associated with an interquartile range increase in PM2.5 (47.5 μg/m3) and PM10 (76.9 μg/m3) concentrations stratified by gender (A), hypertension (B), hyperlipidemia (C) and congestive heart failure (D). **Table S1.** Basic information and numbers of the monitoring sites in each study city. (DOCX 834 kb)

## Abbreviations

CI: Confidence interval
CO: Carbon monoxide
DALY: Disability-adjusted life year
HSR: Hospitalization summary report
ICD-10: International Classification of Diseases, 10th Revision
IQR: Interquartile range
NO_2_: Nitrogen dioxides
OR: Odds ratio
PM: Particle matter
PM_2.5_, PM_10_: Particulate matter ≤2.5-μm (or < 10-μm) in diameter
SO_2_: Sulfur dioxide
