# Supplementary material for: The effect of ambient particle matters on hospital admissions for cardiac arrhythmia: a multi-city case-crossover study in China
Source: Environ Health. 2018 Jul 16;17:60. doi: 10.1186/s12940-018-0404-z (PMC6048711; doi:10.1186/s12940-018-0404-z)
Supplement: Supplementary file 1 — Figure S1. Locations of 26 Chinese cities and their average daily PM2.5 concentration during the study period. 26 Chinese cities included Harbin (71 μg/m3), Changchun (65 μg/m3), Shenyang (72 μg/m3), Dalian(50 μg/m3), Beijing (82 μg/m3), Tianjin (78 μg/m3), Shijiazhuang (106 μg/m3), Jinan (90 μg/m3), Zhengzhou (91 μg/m3), Xi’an (67 μg/m3), Lanzhou (54 μg/m3), Yinchuan (48 μg/m3), Xining (55 μg/m3), Urumchi (64 μg/m3), Chengdu (67 μg/m3), Chongqing (59 μg/m3), Wuhan (75 μg/m3), Changsha (67 μg/m3), Nanchang (46 μg/m3), Nanjing (65 μg/m3), Shanghai (53 μg/m3), Hangzhou (58 μg/m3), Kunming (31 μg/m3), Nanning (45 μg/m3), Guangzhou (43 μg/m3) and Fuzhou (30 μg/m3). Figure S2. Percentage change with 95% confidence interval in arrhythmia admissions associated with an interquartile range increase in PM2.5 (47.5 μg/m3) and PM10 (76.9 μg/m3) concentrations stratified by gender (A), hypertension (B), hyperlipidemia (C) and congestive heart failure (D). Table S1. Basic information and numbers of the monitoring sites in each study city. (DOCX 834 kb) [file 12940_2018_404_MOESM1_ESM.docx]

**Figure S1.** Locations of 26 Chinese cities and their average daily PM_2.5_ concentration during the study period. 26 Chinese cities included Harbin (71μg/m^3^), Changchun (65μg/m^3^), Shenyang (72μg/m^3^), Dalian(50μg/m^3^), Beijing (82μg/m^3^), Tianjin (78μg/m^3^), Shijiazhuang (106μg/m^3^), Jinan (90μg/m^3^), Zhengzhou (91μg/m^3^), Xi’an (67μg/m^3^), Lanzhou (54μg/m^3^), Yinchuan (48μg/m^3^), Xining (55μg/m^3^), Urumchi (64μg/m^3^), Chengdu (67μg/m^3^), Chongqing (59μg/m^3^), Wuhan (75μg/m^3^), Changsha (67μg/m^3^), Nanchang (46μg/m^3^), Nanjing (65μg/m^3^), Shanghai (53μg/m^3^), Hangzhou (58μg/m^3^), Kunming (31μg/m^3^), Nanning (45μg/m^3^), Guangzhou (43μg/m^3^) and Fuzhou (30μg/m^3^).


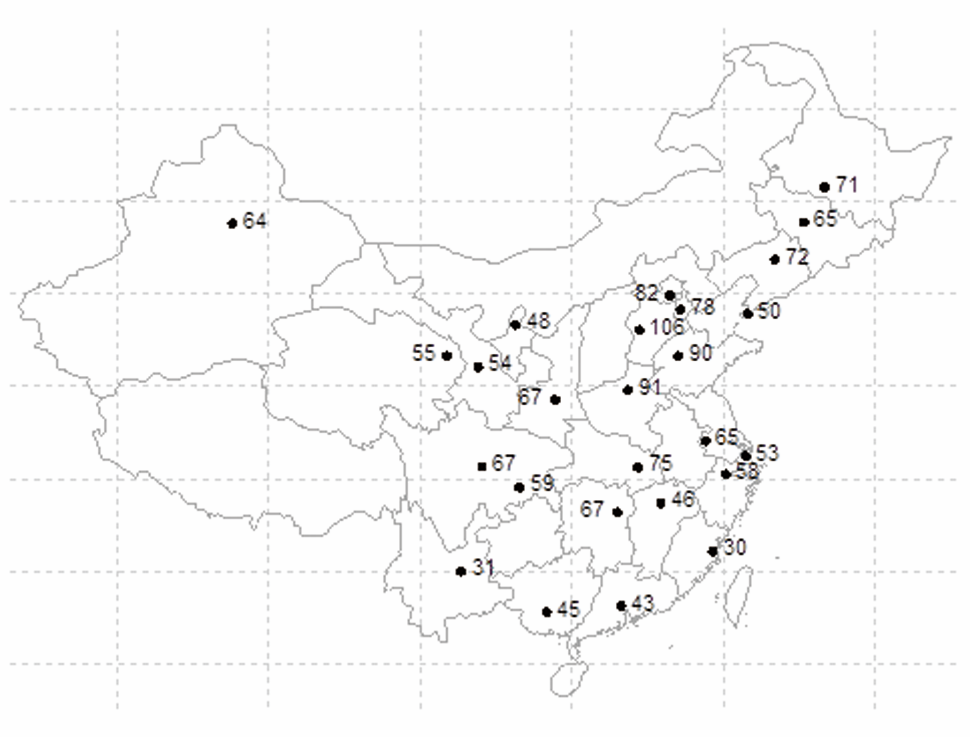


**Figure S2.** Percentage change with 95% confidence interval in arrhythmia admissions associated with an interquartile range increase in PM_2.5_ (47.5μg/m^3^) and PM_10_ (76.9μg/m^3^) concentrations stratified by gender **(A)**, hypertension **(B)**, hyperlipidemia **(C)** and congestive heart failure **(D)**.


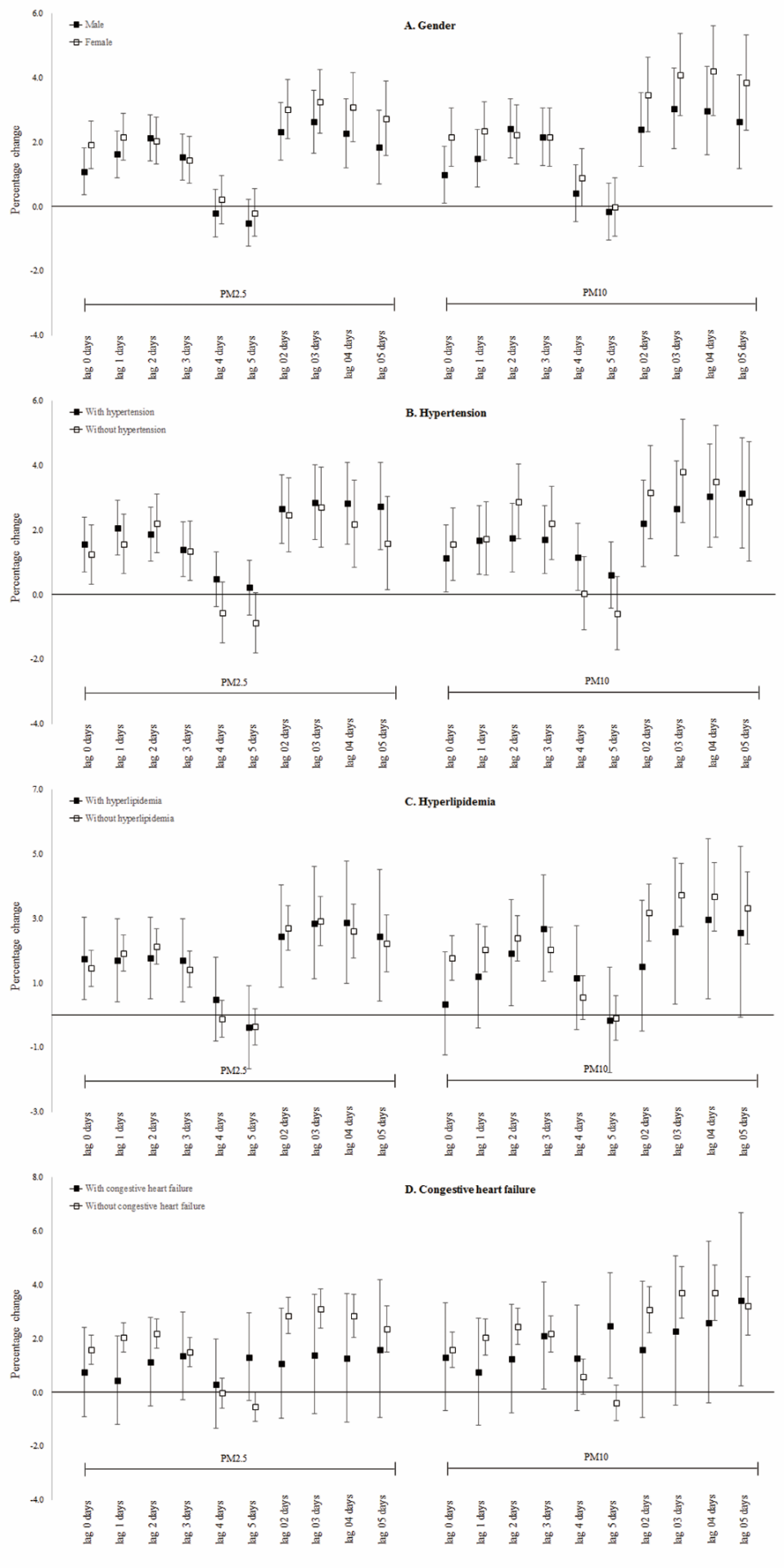


**Detail description of air pollution data**

Data on daily pollutants concentrations for each city was obtained from the National Urban Air Quality Real-time Publishing Platform (<http://106.37.208.233:20035/>). The platform is administrated by the China’s Ministry of Environmental Protection and displays real-time concentrations of criteria air pollutants in all state-controlled monitoring sites. This website provides adequate information for researchers and decision-makers who need to evaluate and analyze the spatial-temporal distribution and variation in air quality in China. The monitoring stations have been designated in a mix of urban and background sites, with most of them in urban areas, and a few in the suburbs and rural areas. These stations are mandated to not be in the direct vicinity of apparent emission sources (eg, traffic, industry, and open burning), so that their measurements can reflect the general urban background level of air pollution. All measurements were made in accordance with China's National Air Quality Control standards (GB3095-2012). Detailed of the station information used in our study were listed below (**Table S1**). A series of standards or regulations have been made on the locations of monitors and the monitoring process of air pollutants by the Chinese government [1], ensuring the monitoring measurements could reflect air pollution levels of urban background [2,3]. The monitoring data has been extensively used as a proxy for population exposure to air pollution in China [3-5].

**Table S1.** Basic information and numbers of the monitoring sites in each study city.

| City | Province | Number of sites in the study city | Latitude | Longitude | Population (Million) |
| --- | --- | --- | --- | --- | --- |
| Harbin | Heilongjiang | 11 | 45.8 | 126.6 | 10.6 |
| Changchun | Jilin | 10 | 43.9 | 125.4 | 7.6 |
| Shenyang | Liaoning | 11 | 41.8 | 123.4 | 7.3 |
| Dalian | Liaoning | 10 | 38.9 | 121.6 | 5.9 |
| Beijing | Beijing | 12 | 39.9 | 116.5 | 21.1 |
| Tianjin | Tianjin | 15 | 39.1 | 117.2 | 14.7 |
| Shijiazhuang | Hebei | 8 | 38.0 | 114.5 | 10.5 |
| Jinan | Shandong | 8 | 36.7 | 117.0 | 6.9 |
| Zhengzhou | Henan | 9 | 34.8 | 113.7 | 9.2 |
| Xi’an | Shaanxi | 13 | 34.3 | 109.0 | 8.6 |
| Lanzhou | Gansu | 5 | 36.0 | 103.7 | 3.6 |
| Yinchuan | Ningxia | 6 | 38.5 | 106.3 | 2.1 |
| Xining | Qinghai | 4 | 36.6 | 101.7 | 2.2 |
| Urumchi | Xinjiang | 7 | 43.8 | 87.7 | 3.3 |
| Chengdu | Sichuan | 8 | 30.7 | 104.1 | 14.3 |
| Chongqing | Chongqing | 17 | 29.6 | 106.5 | 29.7 |
| Wuhan | Hubei | 10 | 30.5 | 114.3 | 10.2 |
| Changsha | Hunan | 10 | 28.2 | 113.0 | 7.1 |
| Nanchang | Jiangxi | 9 | 28.7 | 115.9 | 5.1 |
| Nanjing | Jiangsu | 9 | 32.0 | 118.8 | 8.1 |
| Shanghai | Shanghai | 10 | 31.2 | 121.5 | 24.2 |
| Hangzhou | Zhejiang | 11 | 30.3 | 120.2 | 8.8 |
| Kunming | Yunnan | 7 | 25.0 | 102.7 | 5.4 |
| Nanning | Guangxi | 8 | 22.8 | 108.3 | 6.9 |
| Guangzhou | Guangdong | 11 | 23.2 | 113.2 | 12.9 |
| Fuzhou | Fujian | 6 | 26.1 | 119.3 | 7.2 |

**Reference**

1. Zhao B, Su Y, He S, Zhong M, Cui G (2016) Evolution and comparative assessment of ambient air quality standards in China. Journal of Integrative Environmental Sciences 13: 85-102.

2. Chen R, Samoli E, Wong CM, Huang W, Wang Z, et al. (2012) Associations between short-term exposure to nitrogen dioxide and mortality in 17 Chinese cities: the China Air Pollution and Health Effects Study (CAPES). Environ Int 45: 32-38.

3. Chen R, Kan H, Chen B, Huang W, Bai Z, et al. (2012) Association of particulate air pollution with daily mortality: the China Air Pollution and Health Effects Study. Am J Epidemiol 175: 1173-1181.

4. Chen R, Yin P, Meng X, Liu C, Wang L, et al. (2017) Fine Particulate Air Pollution and Daily Mortality. A Nationwide Analysis in 272 Chinese Cities. Am J Respir Crit Care Med 196: 73-81.

5. Yin P, Chen R, Wang L, Meng X, Liu C, et al. (2017) Ambient Ozone Pollution and Daily Mortality: A Nationwide Study in 272 Chinese Cities. Environ Health Perspect 125.
